# Continuous Superior Trunk Block versus Single-Shot Superior Trunk Block with Intravenous Dexmedetomidine for Postoperative Analgesia in Arthroscopic Shoulder Surgery: A Prospective Randomized Controlled Trial

**DOI:** 10.3390/jcm13071845

**Published:** 2024-03-22

**Authors:** Bora Lee, Jaewon Jang, Joon-Ryul Lim, Eun Jung Kim, Donghu Kim, Yong-Min Chun, Yong Seon Choi

**Affiliations:** 1 Department of Anesthesiology and Pain Medicine, Severance Hospital and Anesthesia and Pain Research Institute, Yonsei University College of Medicine, 50-1 Yonsei-ro, Seoul 03722, Republic of Korea; 2 Department of Orthopedic Surgery, Arthroscopy and Joint Research Institute, Severance Hospital, Yonsei University College of Medicine, Seoul 03722, Republic of Korea

**Keywords:** brachial plexus block, catheters, dexmedetomidine, pain, postoperative, nerve block

## Abstract

**Background/Objectives:** Intravenous dexmedetomidine (DEX) can increase the analgesia duration of peripheral nerve block; however, its effect in combination with superior trunk block (STB) remains unclear. We examined whether combining single-shot STB (SSTB) with intravenous DEX would provide noninferior postoperative analgesia comparable to that provided by continuous STB (CSTB). **Methods:** Ninety-two patients scheduled for elective arthroscopic rotator cuff repair were enrolled in this prospective randomized trial. Patients were randomly assigned to the CSTB or SSTB + DEX group. Postoperatively, each CSTB group patient received 15 mL of 0.5% ropivacaine and a continuous 0.2% ropivacaine infusion. Each SSTB group patient received a 15 mL postoperative bolus injection of 0.5% ropivacaine. DEX was administered at 2 mcg/kg for 30 min post anesthesia, then maintained at 0.5 mcg/kg/h till surgery ended. Pain scores were investigated every 12 h for 48 h post operation, with evaluation of rebound pain incidence and opioid consumption. **Results:** The SSTB + DEX group had significantly higher median pain scores at 12 h post operation (resting pain, 8.0 vs. 3.0; movement pain, 8.0 vs. 5.0) and a higher incidence of rebound pain (56% vs. 20%) than the CSTB group. However, no significant between-group differences were observed in pain scores postoperatively at 24, 36, or 48 h. The CSTB group required less opioids and fewer rescue analgesics within 12–24 h post operation than the SSTB + DEX group. **Conclusions:** Compared with CSTB, SSTB + DEX required additional adjuvant or multimodal analgesics to reduce the risk and intensity of postoperative rebound pain in patients who underwent arthroscopic rotator cuff repair.

## 1. Introduction

Arthroscopic shoulder surgery involves significant pain during the early postoperative period. During this period, postoperative pain control is essential to accomplish early rehabilitation and recovery. Interscalene brachial plexus block (ISB) is the standard approach used to manage acute pain after shoulder surgery. Given that single-shot ISB can provide an analgesic effect for 8 h post operation, studies have reported strategies for optimal pain management, including local anesthetic adjuvants for single-shot or continuous ISB, to control pain beyond 12–24 h after shoulder surgery. Continuous ISB provides better pain relief and less rebound pain than single-shot ISB; however, the procedure necessitates catheter insertion, leading to additional equipment costs and requiring specialized provider training [1,2]. The benefits of ISB may be offset by the related high incidence of hemidiaphragmatic paresis. To spare the phrenic nerve, a superior trunk block (STB) can be performed immediately before the location where the suprascapular nerve branches from the superior trunk. This method provides sufficient analgesia while preserving diaphragmatic function after shoulder arthroplasty [3,4,5]. Most studies have investigated the combined effects of adjuvants with single-shot ISB, with only a few studies comparing the pain control effect of single-shot ISB combined with an adjuvant with that of continuous ISB. The analgesic effects of continuous superior trunk block (CSTB) compared with those of single-shot superior trunk block (SSTB) and intravenous dexmedetomidine (DEX) on pain after arthroscopic shoulder surgery remain unclear.

We hypothesized that SSTB combined with intravenous DEX would provide noninferior analgesia compared with that provided by CSTB in patients undergoing arthroscopic shoulder surgery. This study aimed to examine this hypothesis. The primary study endpoint was pain scores at 24 h post operation. Secondary endpoints included the incidence of rebound pain, opioid consumption, and pain scores at other times within the 48 h postoperative period.

## 2. Materials and Methods

This prospective randomized controlled study was approved by the Severance Hospital Institutional Review Board (protocol number: 4-2021-0853) on 13 August 2021, and registered at ClinicalTrials.gov (NCT05020821, principal investigator: Jaewon Jang, date of registration: 25 August 2021). This study was conducted according to the guidelines of the Declaration of Helsinki and followed the Consolidated Standards for Reporting Trials reporting guidelines for clinical trials. We enrolled 92 adult patients scheduled for elective arthroscopic rotator cuff repair between September 2021 and August 2022. The exclusion criteria were a history of shoulder surgery, contraindications to peripheral nerve block use, allergy to lidocaine or ropivacaine, heart failure, arrhythmia, preoperative bradycardia, opioid use disorder, or hepatic or renal insufficiency. All patients provided written informed consent.

### 2.1. Randomization and Study Protocol

Patients were randomly assigned to the CSTB or SSTB + DEX group using a computer-generated randomization sequence by an investigator not involved in intraoperative patient management. The same investigator provided two sets of solutions, namely, the experimental drug (DEX) and placebo (normal saline), which were both colorless and transparent. The attending anesthesiologists, other investigators, surgeons, and nursing staff were blinded to the group allocation assignments before the nerve block procedure.

A standard monitoring protocol was used after each patient arrived in the operating room. All patients underwent general anesthesia with tracheal intubation using propofol (1.0–2.0 mg/kg), rocuronium (0.6–0.8 mg/kg), and remifentanil (0.05–0.1 mcg/kg/min). Anesthesia was maintained using sevoflurane and remifentanil (0.01–0.1 mcg/kg/min) in an air/oxygen mixture. In the SSTB + DEX group, DEX was administered at 2 mcg/kg for 30 min after anesthesia induction, followed by the administration of the fixed maintenance dose at 0.5 mcg/kg/h until the end of surgery. In the CSTB group, an equivalent volume of the placebo solution was administered similarly. When the heart rate was <50 beats/min or the systolic pressure was <80 mmHg, the anesthesiologist administered an atropine bolus injection (0.5 mg) or a continuous infusion of norepinephrine (0.01–0.1 mcg/kg/min), respectively. Dexamethasone was not administered to exclude its effects as an adjuvant for nerve block. The same surgeon performed all surgical procedures while each patient was in a beach chair position and maintained controlled hypotension. Neuromuscular blockade was reversed using neostigmine (1 mg) and glycopyrrolate (0.2 mg) after the surgical procedure.

### 2.2. Nerve Block Procedure

Nerve blocks were administered postoperatively to avoid catheter dislodgement. The anesthetized patients were placed in a supine position, with the head slightly turned to the contralateral side of the operative side. Subsequently, a linear ultrasound probe (6 to 13-MHz, HFL38xp, SonoSite Inc., Bothell, WA, USA) was used to perform a sequential prescan from the supraclavicular fossa to the upper part of the interscalene groove, and then in the reverse direction to the supraclavicular fossa [6]. After tracing C5 and C6 convergence, we identified the suprascapular nerve originating from the superior trunk. After sterile skin preparation, STB was performed based on the group assignment. In the CSTB group, we used an end-hole perineural catheter through a catheter-over-needle system (E-cath, PAJUNK^®^ GmbH, Geisingen, Germany). An 18-gauge cannula with an indwelling 21-gauge needle was advanced in plane at a lateral-to-medial direction until the tip was located just deep into the superior trunk. Non-adrenalized ropivacaine (10 mL, 0.5%) was injected to facilitate hydrodissection to expose space for catheter insertion. Next, a 21-gauge end-hole E-catheter was inserted through an indwelling 18-gauge cannula. To ensure correct catheter tip placement in the medial aspect beneath the superior trunk, additional non-adrenalized ropivacaine (5 mL, 0.5%) was injected during ultrasound imaging (Figure S1). A sterile occlusive dressing and an anchoring device were used to secure the catheter. In the SSTB + DEX group, STB was performed in a similar manner using an insulated stimulating needle (22 gauge, 50 mm; UniPlex Nanoline, Pajunk, Geisingen, Germany). Local anesthetic (15 mL of 0.5% ropivacaine) with epinephrine (1:200,000) was injected to extensively surround the trunk in the lateral, inferior, and medial aspects.

### 2.3. Postoperative Management

In the post-surgery ward, each patient was prescribed acetaminophen (1200 mg) at 8 h intervals as required to control postoperative pain before hospital discharge. Intravenous tramadol (50 mg) was administered as rescue analgesia when the numeric rating scale pain score (NRS) was >4. No steroids were administered after surgery. In the CSTB group, patients received a continuous infusion of 0.2% ropivacaine (a total volume of 300 mL) at a basal rate of 5 mL/h, a 4 mL bolus, and a 30 min lockout time using a disposable patient-controlled infusion pump (Accufuser, Woo Young Medical, Republic of Korea). In the SSTB + DEX group, DEX was no longer administered after surgery. Based on routine clinical practice, all patients were discharged 1 day post operation. All patients received phone calls from an investigator at 36 and 48 h post operation for evaluation of pain scores and any block- or equipment-related complications. The catheters were removed at each patient’s local clinic 48 h after surgery.

### 2.4. Outcome Assessments

The primary outcome was the 24 h postoperative resting pain score. Pain scores at other times, rebound pain incidence and opioid consumption were included as secondary endpoints. Pain intensities at rest and during activity were assessed using an 11-point numeric pain score (0, no pain, to 10, worst imaginable pain). Pain scores were recorded at five points: the preoperative baseline, and 12, 24, 36, and 48 h postoperatively. Rebound pain was defined as severe pain (NRS score ≥ 7) at the surgical site following the STB resolution. The administration of tramadol was converted to the oral morphine equivalents [7].

### 2.5. Statistical Analysis

The noninferiority hypothesis was used to calculate the sample size based on the primary endpoint. A pain score standard deviation of 3.2 was used for the calculation [8]. The predetermined noninferiority margin was 2 points on the 11-point pain intensity scale [9]. To achieve a significance level of 2.5% and power of 80%, 41 patients were required in each group. To account for a dropout rate of 10%, 46 patients were included in each group. The noninferiority hypothesis for the primary outcome was assessed using a one-sided *t*-test (between-group difference in pain score of ≥2 points) at a significance level of 2.5%. We performed between-group comparisons of preoperative measurements and secondary outcomes. Parametricity was confirmed using the Shapiro–Wilk and Kolmogorov–Smirnov tests. Parametric and non-parametric continuous variables were analyzed using an independent *t*-test and a Mann–Whitney U test, respectively. Between-group comparisons of categorical variables were conducted using Fisher’s exact test or a χ^2^ test, as appropriate. Parametric and non-parametric continuous variables are shown as means ± standard deviation and medians (interquartile range), respectively, while categorical variables are shown as numbers (percentages). All statistical analyses were performed using R (version 3.5.1; R Foundation for Statistical Computing, Vienna, Austria), IBM SPSS Statistics for Windows, version 23.0 (IBM Corp., Armonk, NY, USA), or MedCalc Statistical Software version 18.11.3. Statistical significance was set at *p* < 0.05.

## 3. Results

**Figure 1:**
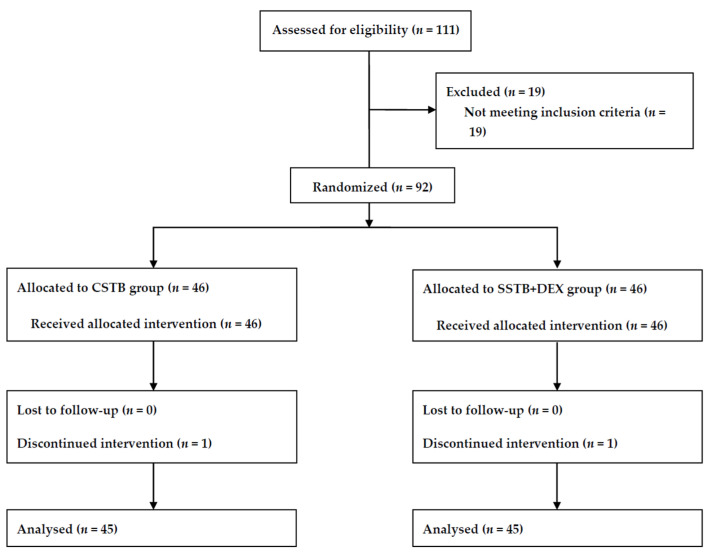
CONSORT study flow diagram. CONSORT, Consolidated Standards for Reporting Trials; CSTB, continuous superior trunk block; SSTB + DEX, single shot of superior trunk block with intravenous dexmedetomidine.

Among the 111 patients screened for eligibility, we enrolled 92 and allocated them to either group. One CSTB group patient was excluded because her Horner’s syndrome diagnosis required catheter removal at 6 h post operation. One SSTB + DEX group patient was excluded due to changes in the surgical procedure. Accordingly, we included data from 90 patients in the final analysis. A flowchart of the study is presented in Figure 1.

No significant between-group differences were observed in patient characteristics and operative data (Table 1). No significant between-group differences were found in the number of patients requiring intraoperative atropine, the fluid amount, or the minimum blood pressure; however, the minimum heart rate was significantly lower in the SSTB + DEX group than in the CSTB group (49 bpm vs. 52 bpm, *p* = 0.007). In addition, systolic, mean, and diastolic blood pressures were lower in the CSTB group compared with that of SSTB + DEX group, but without statistical significance.

**Figure 2:**
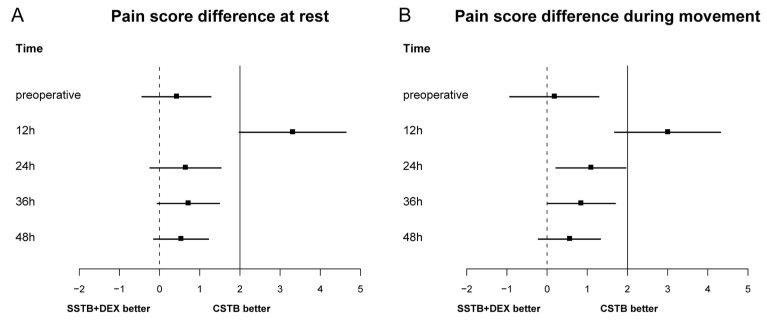
Noninferiority diagram of between-group differences in the numerical rating scale pain scores within 48 h post operation, both at rest (**A**) and during movement (**B**). The solid line indicates a noninferiority margin (δ) of 2. Squares indicate differences in the mean pain score, while error bars indicate the 95% CIs of the between-group differences. Statistical significance was set at *p* < 0.05. CSTB, continuous superior trunk block; SSTB + DEX, single shot of superior trunk block with intravenous dexmedetomidine.

**Figure 3:**
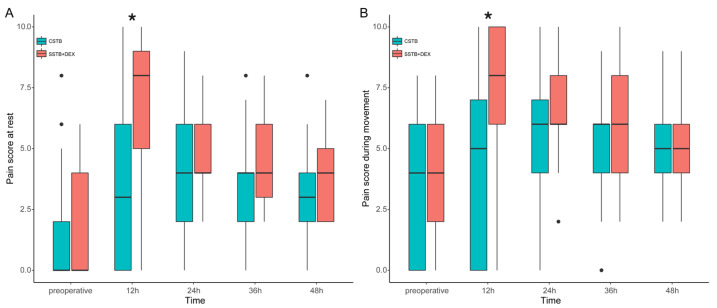
Pain scores at rest (**A**) and during movement (**B**). Boxplot represents the median with the 25th/75th percentile. Whiskers reveal the minimum/maximum values, excluding outliers. Points represent the outliers. CSTB, continuous superior trunk block; SSTB + DEX, single shot of superior trunk block with intravenous dexmedetomidine. * *p* < 0.05 between the CSTB and SSTB + DEX groups in the post hoc analysis.

The mean scores for resting pain 24 h post operation were 4.4 ± 2.3 and 5.0 ± 2.0 in the CSTB and SSTB + DEX groups, respectively, and the between-group difference was 0.64 (95% confidence interval [CI], −0.25 to 1.54). Since the upper limit of the 95% CI was lower than the predefined noninferiority margin (*δ* = 2), noninferiority was established (Figure 2). The mean difference in the moving pain score 24 h post operation was 1.09 (95% CI, 0.21–1.97), which also indicated noninferiority. Similarly, the lack of a significant between-group difference in the resting and moving pain scores 36 and 48 h postoperatively also indicated noninferiority. However, the resting and moving pain scores at 12 h post operation were significantly higher in the STB + DEX group than in the CSTB group, with a mean between-group difference of >2 (mean difference in resting pain score, 3.31; 95% CI, 1.97 to 4.65; mean difference of moving pain score, 3; 95% CI, 1.67–4.33). The pain scores at 12 h post operation indicated that SSTB + DEX was inferior to CSTB. The resting and moving median pain scores at 12 h post operation were significantly higher in the SSTB + DEX group than in the CSTB group (resting pain, 8.0 vs. 3.0; movement pain, 8.0 vs. 5.0), with no between-group difference in the pain scores at the other time points (Figure 3).

The median between-group time difference in time to first pain was 3 h (14 h vs. 11 h, *p* = 0.014, Table 2). The rebound pain incidence was 20% and 56% in the CSTB and SSTB + DEX groups, respectively (*p* = 0.001); the overall incidence was 38%. Opioid consumption within the 12–24 h postoperative period was higher in the SSTB + DEX group than that in the CSTB group. The SSTB + DEX group had more patients requiring rescue analgesics within the 12–24 h post operation than the CSTB group. DEX use was not associated with a prolonged post-anesthesia care unit stay. No patients experienced adverse pulmonary events and, at the 48 h follow-up, no patients reported any block- or equipment-related complications.

## 4. Discussion

In our study, SSTB + DEX yielded significantly inferior analgesia at 12 h post operation than CSTB did. Furthermore, SSTB was associated with a higher rebound pain incidence than CSTB. The patients in the CSTB group required less opioid consumption and fewer rescue analgesics within the postoperative 12–24 h period than those in the SSTB + DEX group. However, SSTB + DEX yielded pain scores comparable to those of CSTB within 24–48 h post operation.

Single-injection ISB is an important component of multimodal analgesia for postoperative pain and provides effective analgesia during the early postoperative period (6–8 h postoperatively) to reduce opioid requirement [10]. Continuous ISB has been shown to lead to better analgesia, less opioid consumption, better sleep patterns, and a higher quality of recovery than single-injection ISB [11,12,13]. Given the difficulty and cost of catheterization, most recent studies have focused on increasing the analgesic duration of single-shot ISB by adding perineural or intravenous adjuvants [14,15,16,17,18]. Among the various adjuvants for local anesthetics, intravenous DEX prolongs the analgesic duration of single-shot ISB and reduces postoperative analgesic consumption [3,19,20]. However, limited studies exist on the effectiveness of intravenous administration of DEX, in addition to STB, a phrenic-sparing alternative to ISB. Therefore, this study compared the postoperative analgesic effect of CSTB with that of combined SSTB and intravenous DEX in patients undergoing arthroscopic rotator cuff repair.

DEX provides prolonged analgesia when used as a single-shot peripheral nerve block adjuvant [18,19,20,21]. In contrast, perineural DEX delays rehabilitation due to prolonged motor blockade [21]. Its use includes specific potential risks, including “off-label” use in the absence of US Food and Drug Administration approval. A recent study showed that intravenous dexamethasone and DEX notably extended the period before the first pain relief request after single-shot ISB following arthroscopic shoulder surgery [17]. Intravenous DEX (2.0 mcg/kg) significantly increases the ISB analgesia duration (median increase of 218 min, compared with the control group). It also reduces the cumulative opioid consumption within the 24 h postoperative period [14]. Kang et al. revealed that 0.5 mcg/kg or 1.0 mcg/kg of intravenous DEX did not have a clinical analgesic effect, with only 2 mcg/kg of intravenous DEX significantly increasing the duration of ISB [14]. Accordingly, we administered a 2 mcg/kg loading dose of intravenous DEX after induction, followed by continuous intravenous infusion of a maintenance dose of 0.5 mcg/kg/h until the end of surgery [18,22]. Central nerve system *α*2 receptor binding mediates the analgesic effect of intravenous DEX. Pain transmission is suppressed via subdued interneuron hyperpolarization and inhibition of nociceptive transmitter (e.g., substance P and glutamate) release [14,17,22].

Generally, pain after arthroscopy peaks on postoperative day 1, with severe postoperative pain occurring in some cases within the first 48 h post operation [23]. Here, the patients in the CSTB group had lower opioid consumption than those in the SSTB + DEX group within 12–24 h post operation, which is consistent with previous reports [8,11,13,24,25]. This could be explained by rebound pain after STB resolution. Although single-shot peripheral nerve block is an effective analgesic method, patients can experience a relatively rapid increase in severe pain following block resolution, which is referred to as rebound pain [26]. Since brachial plexus block or STB completely blocks the surgical site for shoulder surgery, there is a higher risk of rebound pain after the block wears off following shoulder surgery than following other surgeries, including total knee arthroplasty [26]. In the absence of adequate systemic analgesia after block resolution, this rebound pain can be attributed to unmasking the expected nociceptive response. Strategies for reducing rebound pain include routine prescription of a systemic multimodal analgesic regimen and extending the analgesic duration of peripheral nerve block through the continuous catheter technique or local anesthetic adjuvants [2]. The multimodal analgesic regimen frequently included acetaminophen, nonsteroidal anti-inflammatory medications, and gabapentinoids to reduce opioid consumption [27]. The rebound pain is associated with age, female sex, bone surgery, and the absence of intraoperative dexamethasone [26]. Perineural dexamethasone adjunct to single-shot ISB decreases the incidence and severity of rebound pain (37% vs. 83%), as well as sleep disturbance, compared with a placebo [15]. Here, the patients in the CSTB group exhibited a significantly lower incidence of rebound pain than those in the SSTB + DEX group (20% vs. 56%). Our study found that the rate of rebound pain within the CSTB group was markedly lower than that seen in previous research, where perineural dexamethasone was used as an adjuvant to single-shot ISB; however, the study designs of the previous and the present study were not identical and cannot be directly compared [15]. Our study revealed that patients who received SSTB + DEX required more analgesics to reduce the intensity of rebound pain within 12–24 h post operation, which implies that individuals in the SSTB + DEX group need an additional multimodal analgesic regimen to reduce the rebound pain compared to those in the CSTB group in patients undergoing arthroscopic rotator cuff repair. Most studies on single-shot ISB using DEX [14,17] assessed pain occurring within 24 h post operation while not considering the occurrence of rebound pain. Furthermore, unlike our study, the previous studies included not only rotator cuff repair but also Bankart repair, which has a lower postoperative pain intensity [14,17]. Therefore, further studies are warranted to determine whether DEX can effectively reduce rebound pain as dexamethasone can after arthroscopic rotator cuff repair. Additionally, we compared the hemodynamic differences between the two groups. The influence of DEX resulted in a statistically significantly lower minimum heart rate in the SSTB + DEX group compared with the CSTB group. Conversely, systolic, diastolic, and mean blood pressures tended to be higher in the SSTB + DEX group. Moreover, although not statistically significant, the amount of norepinephrine administered was higher in the CSTB group. This is thought to occur due to the reduced use of other anesthetics for maintaining anesthesia or biphasic blood pressure responses as a result of DEX administration [28]. The initial doses of DEX caused a 13% decrease in mean arterial pressure, while subsequent higher doses led to a gradual rise in mean arterial pressure, with peak individual increases averaging 12% above the baseline [28]. Using IV DEX as an adjuvant, considering the risk of bradycardia induced by DEX, appears necessary.

This study had some limitations. Firstly, blinding was not optimal since patients in the SSTB + DEX group were not provided with sham catheters. To avoid investigator bias, neck dressings were adequately concealed in all patients until they were discharged. Secondly, SSTB has been associated with significantly less hemidiaphragmatic paresis than single-shot ISB [3]. We did not compare whether CSTB caused more hemidiaphragmatic paresis than SSTB due to accumulated local anesthetic volume. However, none of our patients experienced postoperative dyspnea or desaturation. Thirdly, we did not use other adjuvants, including dexamethasone, to investigate the effect of DEX. Therefore, additional studies are needed to determine whether the simultaneous use of DEX and dexamethasone in addition to STB can effectively reduce rebound pain, especially after arthroscopic rotator cuff repair. Finally, the generalizability of this study to patients in clinical settings different from those in our center, such as outpatients or patients using strong opioids, may be limited.

## 5. Conclusions

CSTB had lower pain scores at 12 h post operation and was associated with a lower incidence of rebound pain and opioid consumption than SSTB + DEX. SSTB + DEX requires more systemic multimodal analgesics or adjuvants to reduce the risk and intensity of rebound pain within 12–24 h post operation and there was also an additional risk of DEX-induced bradycardia.

## Figures and Tables

**Table 1:** Demographic and operative data.

	CSTB Group (*n* = 45)	SSTB + DEX Group (*n* = 45)	*p* Value
Age (years)	65 ± 7	62 ± 8	0.126
Female/Male	20/25	19/26	>0.999
Height (cm)	160.6 ± 8.5	160.8 ± 8.9	0.931
Weight (kg)	64.5 (59.0–70.5)	62.5 (57.0–69.0)	0.345
Body mass index (kg/m^2^)	24.9 (23.5–26.2)	24.6 (22.8–26.1)	0.399
ASA class (I/II/III)	22/16/7	17/25/3	0.121
Surgical procedure			
Arthroscopic rotator cuff repair	45 (100)	45 (100)	
Subpectoral biceps tenodesis	30 (67)	22 (49)	0.135
Operation time (min)	90 (70–115)	90 (70–105)	0.539
Anesthesia time (min)	148.7 ± 29.9	152.9 ± 31.4	0.515
Fluid amount (mL)	550 (450–650)	550 (450–650)	0.939
Remifentanil use (mcg)	448 (388–576)	442 (371–579)	0.473
Norepinephrine (mcg)	256 (117–336)	208 (124–293)	0.416
The number of patients who required intraoperative atropine	0	3 (7)	0.242
Minimum SBP	81 (78–83)	83 (78–85)	0.256
Minimum DBP	36 ± 6	38 ± 6	0.067
Minimum MBP	52 (49–57)	55 (51–59)	0.088
Minimum heart rate	52 (49–57)	49 (46–53)	0.007

Values are presented as median (interquartile range), mean ± standard deviation, or number of patients (%). ASA, American Society of Anesthesiologists; CSTB, continuous superior trunk block; DBP, diastolic blood pressure; MBP, mean blood pressure; SBP, systolic blood pressure; SSTB + DEX, single shot of superior trunk block with intravenous dexmedetomidine.

**Table 2:** Postoperative variables.

	CSTB Group (*n* = 45)	SSTB + DEX Group (*n* = 45)	*p* Value
Time to first pain report (h)	14 (10–15)	11 (10–13)	0.014
Patients experiencing rebound pain (*n*)	9 (20)	25 (56)	0.001
Patients requiring rescue analgesics (*n*)			
0–12 h	9 (20)	9 (20)	>0.999
12–24 h	23 (51)	36 (80)	0.008
Opioid consumption (morphine equivalents)			
0–12 h	0 (0–0)	0 (0–0)	>0.999
12–24 h	10 (0–10)	20 (10–20)	<0.001
Postoperative anesthesia care unit (PACU) stay (min)	38 (30–55)	48 (35–63)	0.070

Values are presented as median (interquartile range) or number of patients (%). CSTB, continuous superior trunk block; SSTB + DEX, single shot of superior trunk block with intravenous dexmedetomidine.

## Data Availability

The data that contribute to the findings of this study can be obtained from the corresponding author upon reasonable request.

## Supplementary Materials

The following supporting information can be downloaded at: https://www.mdpi.com/article/10.3390/jcm13071845/s1, Figure S1: Ultrasonographic image of the catheter for superior trunk block (blue arrowheads). ASM, anterior scalene muscle; MSM, middle scalene muscle; SA, subclavian artery; SSN, suprascapular nerve; ST, superior trunk.
