# Supplementary figures and images for: Continuous Superior Trunk Block versus Single-Shot Superior Trunk Block with Intravenous Dexmedetomidine for Postoperative Analgesia in Arthroscopic Shoulder Surgery: A Prospective Randomized Controlled Trial
Source: J Clin Med. 2024 Mar 22;13(7):1845. doi: 10.3390/jcm13071845 (PMC11012364; doi:10.3390/jcm13071845)

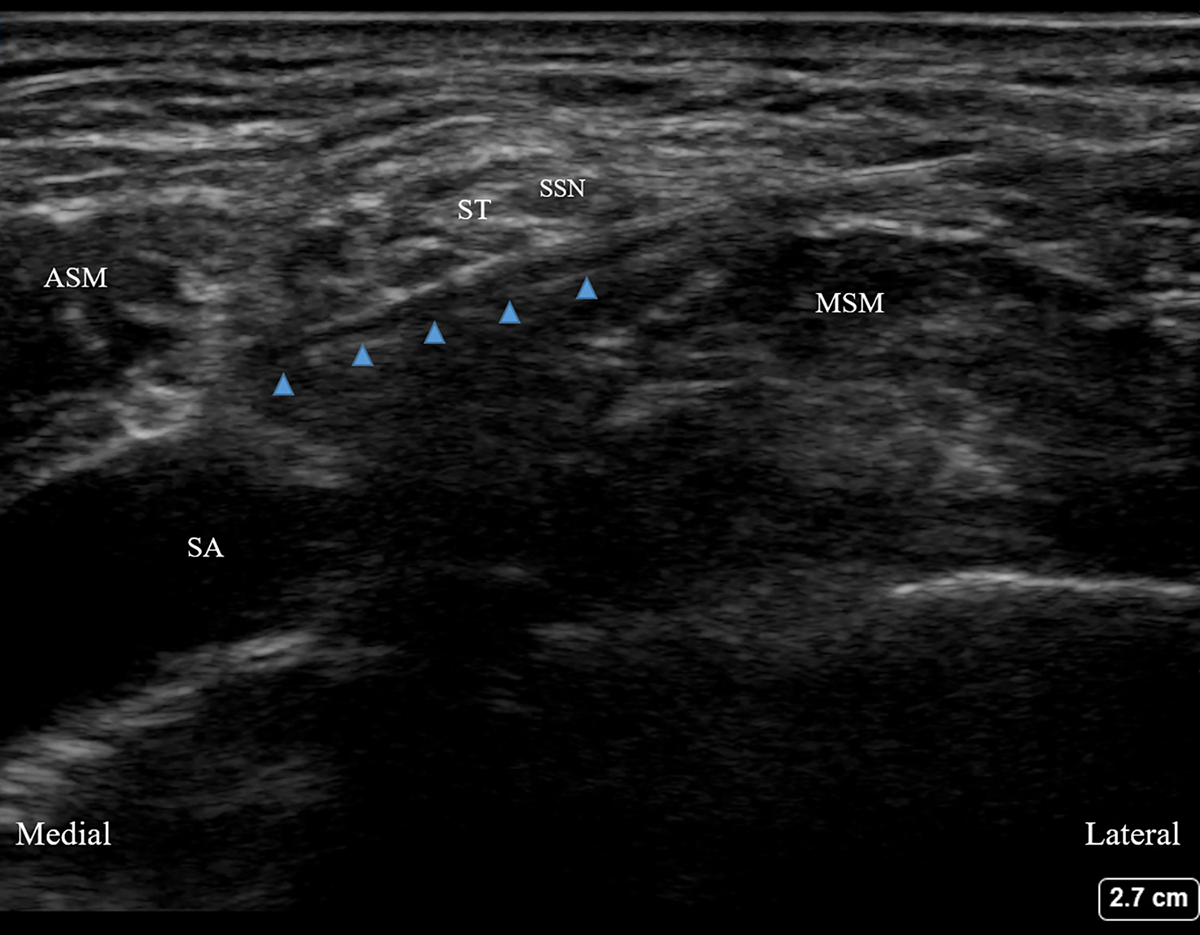

Supplement: Supplementary file 1 [file jcm-13-01845-s001.zip › jcm-2894256-supplementary.tif]
